# Case report: Simultaneous resections of pulmonary segment and an esophageal leiomyoma during spontaneous ventilation video-assisted thoracoscopic surgery

**DOI:** 10.3389/fonc.2024.1364306

**Published:** 2024-05-21

**Authors:** Yi Ding, Lei Shan, Peichao Li, Ning Li, He Zhang, Bo Cong, Hua Zhang, Zhongxian Tian, Xiaogang Zhao, Yunpeng Zhao

**Affiliations:** ^1^ Department of Thoracic Surgery, The Second Hospital of Shandong University, Jinan, China; ^2^ Department of Anesthesiology, The Second Hospital of Shandong University, Jinan, China; ^3^ Department of Thoracic Surgery, Shandong Public Health Clinical Center of Shandong University, Jinan, China

**Keywords:** simultaneous resections, spontaneous ventilation video-assisted thoracoscopic surgery, pulmonary segment, esophageal leiomyoma, thoracoscopic surgery

## Abstract

Spontaneous ventilation video-assisted thoracoscopic surgery (SV-VATS) has rapidly developed in recent years. The application scope is still being continuously explored. We describe a case in which a 40-year-old woman with mixed ground-glass opacity (GGO) and an esophageal leiomyoma successfully underwent simultaneous segmentectomy and leiomyoma resection through spontaneous ventilation video-assisted thoracoscopic surgery. The perioperative course was uneventful. Postoperative pathology revealed minimally invasive adenocarcinoma and esophageal leiomyoma.

## Introduction

Esophageal leiomyoma is considered to be the most common benign esophageal tumor (1). Minimally invasive surgery has become increasingly common in the treatment of esophageal leiomyoma, and a meta-analysis revealed that video-assisted thoracoscopic surgery (VATS) was superior to thoracotomy (2). Pulmonary segmentectomy may be performed to treat early non-small cell lung cancer (NSCLC). A recent randomized controlled trial, JCOG0802, compared the efficacy of segmentectomy and lobectomy and demonstrated that segmentectomy is an effective treatment for stage Ia NSCLC (3). Pulmonary surgery has also been safely performed through spontaneous ventilation video-assisted thoracoscopic surgery (SV-VATS), which is advantageous in that it avoids complications associated with general anesthesia and intubation (4). We report the simultaneous resection of the right S2 and an esophageal leiomyoma during SV-VATS.

## Case report

**Figure 1:**
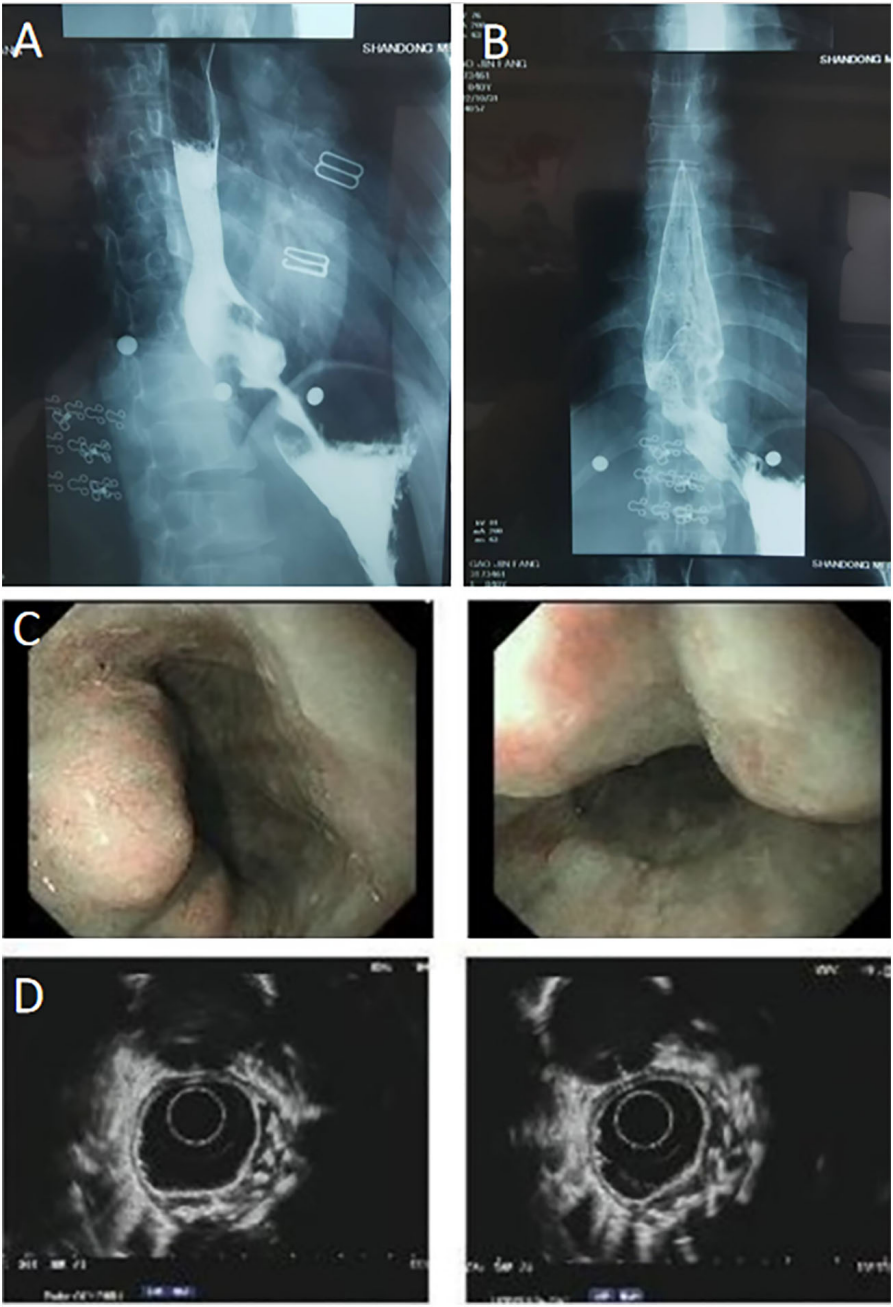
**(A, B)** Preoperative esophageal barium meal examination revealed the presence of an esophageal occupied-lesion. **(C, D)** Endoscopy also indicates the presence of a submucosal esophageal occupied-lesion. Chronic non-atrophic gastritis was also found.

**Figure 2:**
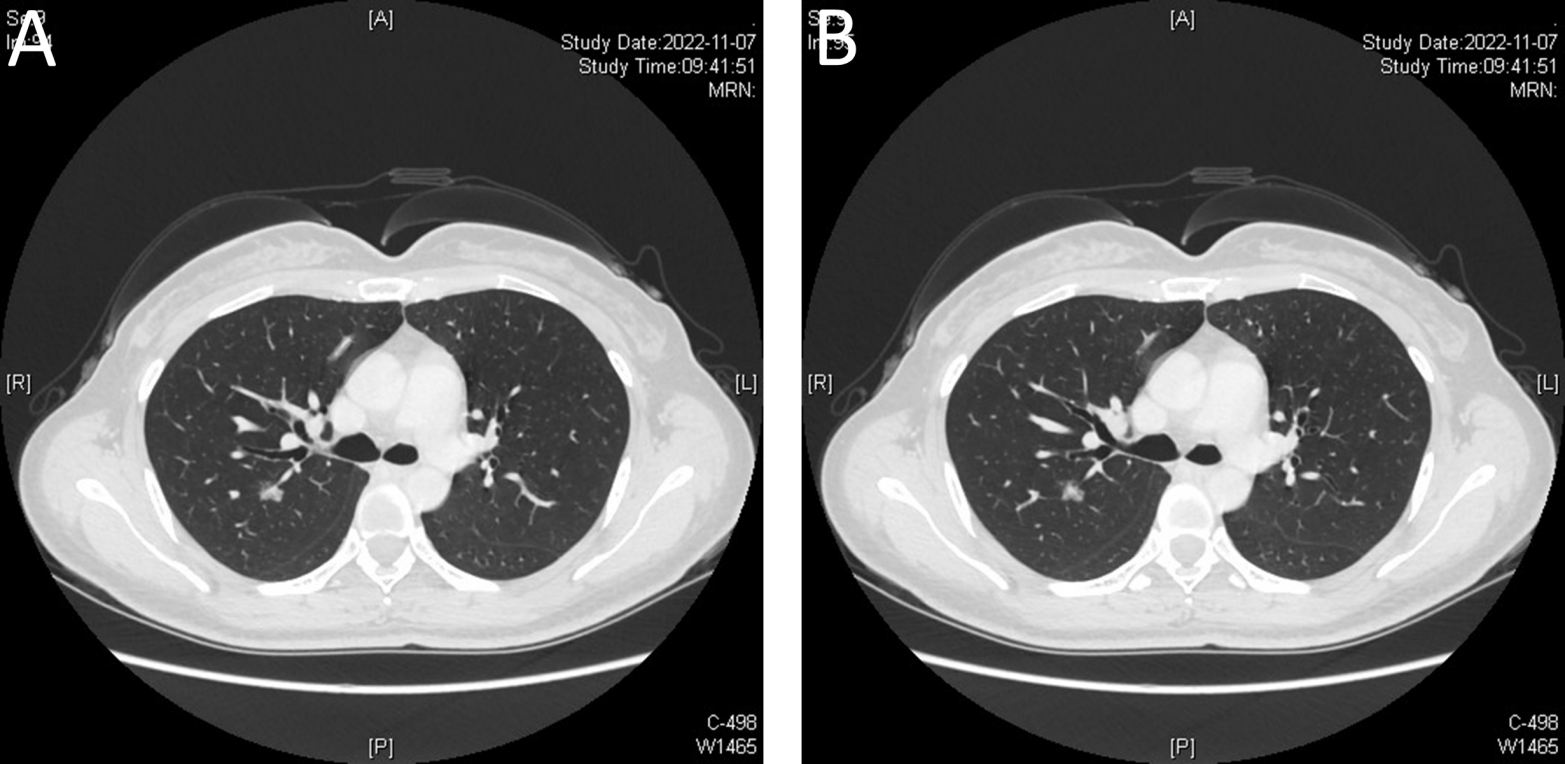
**(A, B)** Enhanced computed tomography (CT) indicates a mixed ground glass opacity. Blood vessels can be seen passing through the lesion, and the enhanced scan shows that the solid components of the lesion are few, and the degree of enhancement is unclear.

In this brief report, we present the case of a 40-year-old woman with GGOs and an esophageal leiomyoma. GGOs in the right upper lobe were discovered eight weeks prior due to a health checkup, and the patient was placed under surveillance. One week prior, an esophageal lesion was found during barium meal examination (**Figures 1A, B**), and endoscopy was performed 3 days later (**Figures 1C, D**). The enhanced computed tomography (CT) scan after admission showed a mixed ground glass opacity the posterior segment (S2) of the right upper lobe, with a maximum cross-section of approximately 1.3 x 1.1 cm (**Figures 2A, B**). Compared with the state of the nodule 8 weeks prior, no change was detected. No clear boundary was observed between the esophagus and the localized soft tissue observed in the lower part of the thoracic esophagus. Mildly homogeneous enhancement was observed in the local mass. Preoperative planning included 3D simulation of the pulmonary structures for segmentectomy. After the preoperative evaluation and multidisciplinary treatment (MDT) discussion, the patient, with a low body mass index (BMI) of 20.2 (height of 165 cm, weight of 55 kg), decided to undergo nonintubated surgery. Patients who undergo nonintubated surgery might have a faster postoperative recovery, lower complication rates, and lower stress hormone levels.

**Figure 3:**
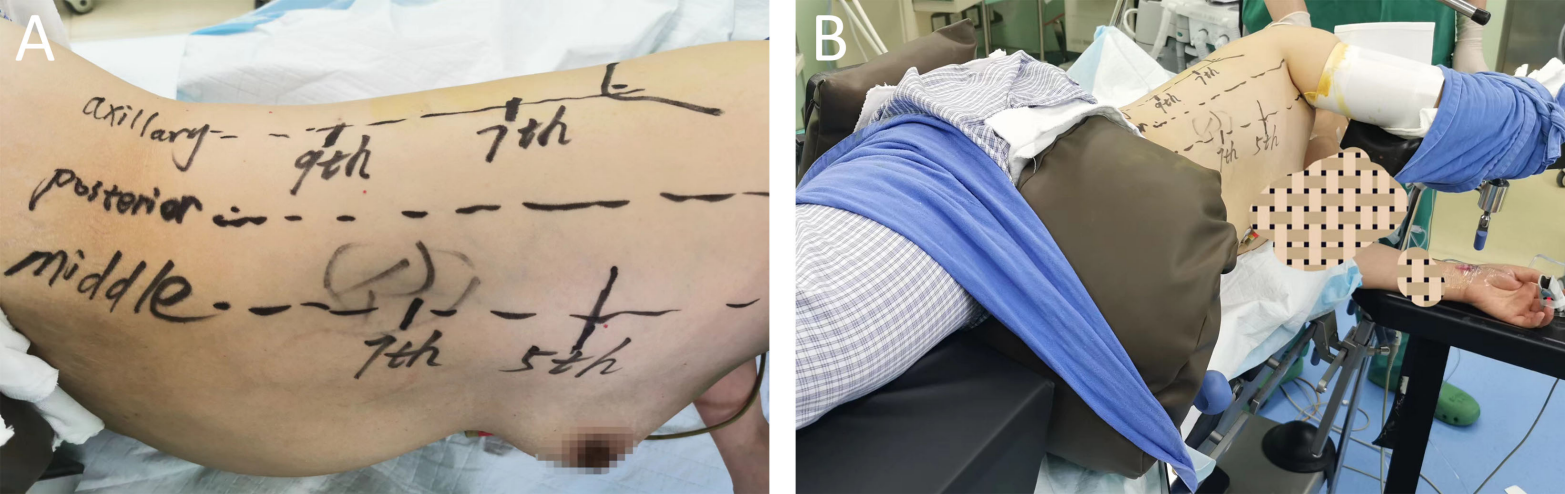
**(A, B)** The patient’s positioning on the operating room bed with all the chest accesses.

The nonintubated approach is performed by an anesthesiologist who is well trained and experienced in performing SV-VATS (5). Intravenous anesthesia combined with local intercostal nerve block was used. The patient was placed in the left dorso-lateral position (6, 7) (**Figures 3A, B**). The first incision was made at the 5th intercostal space in the right mid-axillary line. Then, the visceral pleural surface was anesthetized, an intercostal nerve block at T2-T11 and the thoracic vagus nerve was performed by the surgeon through the incision made in the 5th intercostal space (transient uniport status). Then, the 7th intercostal space was incised along the right mid-axillary line (thoracoscopic observation hole), and the 9th intercostal space was incised along the posterior axillary line for segmentectomy.

First, the oblique fissure was separated, and then the ascending A2 was exposed, ligated and cut. Then, B2 was identified and cut using a stapler. The intersegmental plane was separated along the inflation-deflation line using a stapler when the segmental hilum could be sufficiently lifted. The specimen was sent for frozen sectioning. Minimally invasive adenocarcinoma (MIA) was reported 30 minutes later. The mediastinal lymph nodes of Group 2/4 and Group 7 were sampled.

Then, the esophageal leiomyoma was removed during thoracoscopic surgery. A port was added along the posterior axillary line of the 7th intercostal space. The posterior mediastinal pleura was opened above and below the mass. A sufficient portion of the esophagus was exposed. The lower thoracic esophagus was enlarged, hard, and bulging. A longitudinal myotomy was performed over the mass. Although the outer muscle layer of the leiomyoma was peeled off, it was difficult to expose the entire irregularly shaped mass. A hook cautery and cottonoid sponge were used to carefully separate the mass from the submucosa. The circumference of the lower part of the esophagus was checked to ensure complete removal of the leiomyoma. A water injection test using a nasogastric tube revealed no leakage. The muscular layers were sutured. The entire surgical process took approximately 90 minutes and was performed through SV-VATS (**Supplementary Video 1**).

**Figure 4:**
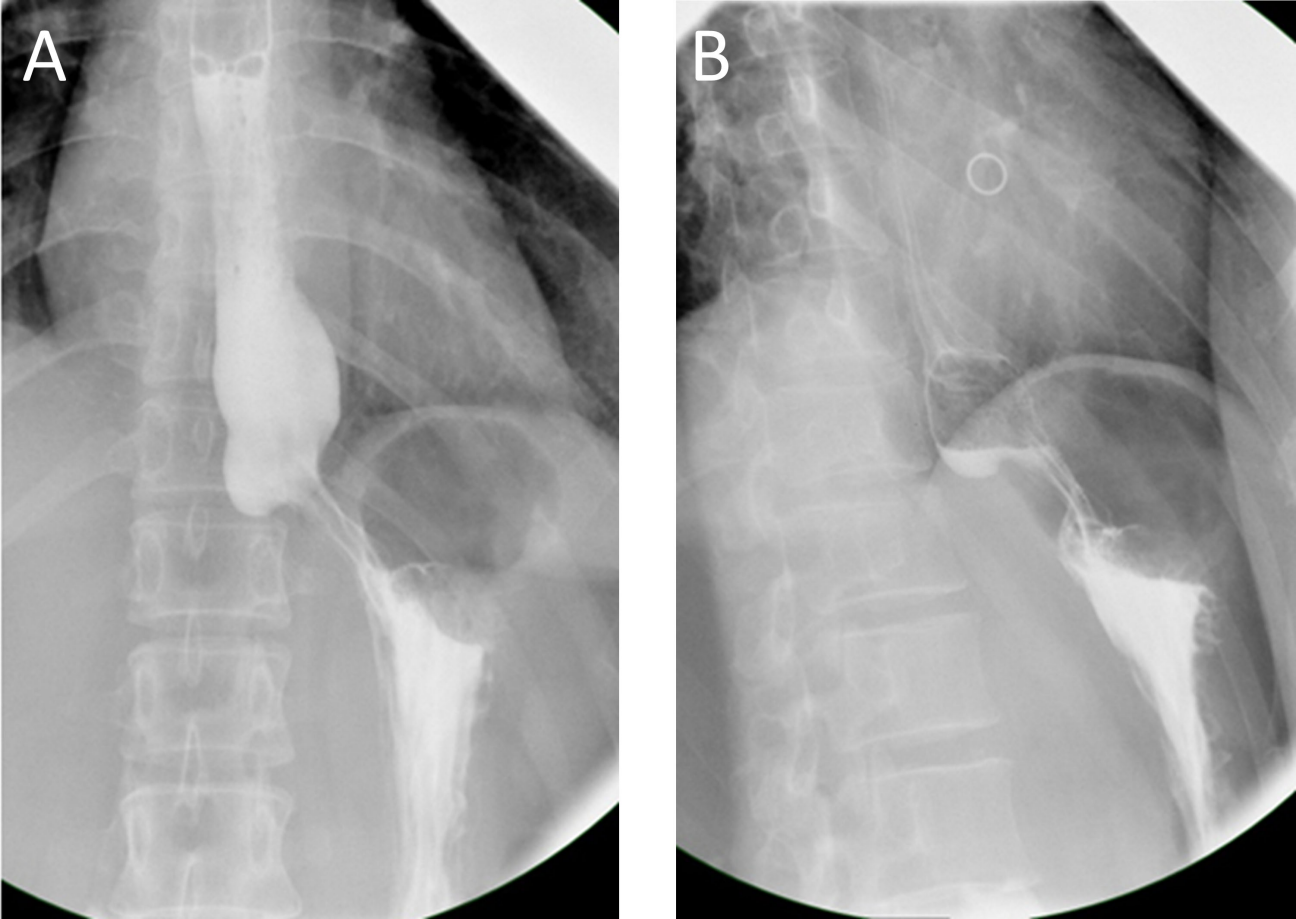
**(A, B)** No esophageal leakage was found during the barium swallow.

This patient’s postoperative course was uneventful. Both the barium swallow (**Figures 4A, B**) and methylthionine chloride swallow were negative for esophageal leakage. The chest drain was removed on postoperative day 2, and the patient was discharged on postoperative day 3. The final pathological report revealed a 7 cm x 4 cm x 3 cm benign leiomyoma and MIA in the upper lobe of the right lung, with negative lymph nodes. Three months after surgery, CT was performed, and no abnormalities were found (**Supplementary Videos 2**, **3**).

## Discussion

Nonintubated uniportal VATS is less invasive and has been shown to reduce the incidence of postoperative complications, shorten the length of hospital stays and reduce the risk of perioperative mortality (8). The same short-term and long-term prognostic outcomes have been reported (9) for nonintubated uniportal VATS and mechanical ventilation VATS (MV-VATS), which is feasible and safe for the treatment of non-small cell lung cancer (NSCLC) in patients with poor lung function. It was even reported that for patients with invasive NSCLC, SV-VATS lobectomy had better long-term outcomes than MV-VATS (10). Patients who underwent subxiphoid SV-VATS thymectomy also recovered quickly and were discharged from the hospital early (11, 12). In surgical lung biopsy, nonintubated VATS is safe and effective and can be used as an effective alternative to standard VATS, even in obese patients (13, 14). Anesthesiological and surgical approach should be reserved in the institution with a highly specialized team. Compared to standard surgery, SV-VATS is more challenging. Not only to develop a rigorous protocol, but also pay close attention to the patient, while requiring the close cooperation of the entire team. We have performed hundreds of SV-VATS procedures for pulmonary wedge resection, segmentectomy, lobectomy, and subxiphoid thymectomy, and surgeons have mastered the procedure.

SV-VATS uniport segmentectomy is routinely performed at our center, and indocyanine green (ICG) is used for fluorography when the inflation−deflation line is not clear. In this case, GGOs in the S2 and an esophageal leiomyoma were discovered simultaneously, and as a result, we performed surgery under SV-VATS. However, no corresponding literature on the simultaneous resection of the pulmonary segment and an esophageal leiomyoma under SV-VATS has been published. A multidisciplinary team including the Thoracic Surgery Department, Anesthesiology Department, and Gastroenterology Department was organized to discuss this case. We have developed precise protocols based on expert consensus (5), including indicators, perioperative management, anesthesia, and surgical procedures. The risk response scheme, anesthesia management strategies and surgical details were also included in the preoperative plan. We believe that simultaneous resection of pulmonary segments and esophageal leiomyomas is safe and feasible (15, 16). Fortunately, the surgery was successful, and the patient recovered rapidly without complications.

Both surgeons and anesthesiologists are concerned about the unexpected occurrence of intraoperative events during SV-VATS, such as massive or fatal bleeding or respiratory and circulatory instability. If necessary, nonintubated surgery should be quickly converted to intubated surgery to avoid catastrophic situations (8, 17). Some anesthesia techniques could also be used to alleviate adverse reactions (18) during nonintubated surgery. SV-VATS is demanding for surgeons and anesthesiologists. The anesthesiologist is always alerted to changes in vital signs so as to make appropriate and timely adjustments. Compared to intubated patients under general anesthesia, nonintubated patients have significantly less tissue damage and lower levels of proinflammatory cytokines. Nonintubated anesthesia could still ensure the stability of circulation and respiration during VATS and facilitate better recovery (19). However, severe mediastinal flutter affects the safety of surgery and may not be beneficial for patients, and appropriate indications still need to be explored.

Nonintubated VATS has only been performed for approximately 20 years. In 2004, Pompeo et al. (20) demonstrated that nonintubated VATS is feasible. In 2007, Al-Abdulatief et al. (21) reported that nonintubated major thoracic surgery is feasible. Since then, research on nonintubated surgery has become more intensive, and surgical procedures have become more complex. Thoracoscopic surgery under spontaneous ventilation is also feasible for tracheal and carinal resection and reconstruction (22). In recent studies, the use of RATS in tracheal/airway surgery in combination with nonintubated spontaneous ventilation has been shown to be safe and feasible (23). With improvements in anesthesia management and surgical skills, nonintubated surgery is constantly improving.

This is the case of a patient who required simultaneous resection of lung cancer and an esophageal leiomyoma, although not previously reported, SV-VATS could also be used for simultaneous resection of pulmonary segments and esophageal leiomyomas. Our case report may provide some reference for those willing to implement this technique.

## Data availability statement

The original contributions presented in the study are included in the article/**Supplementary Material**. Further inquiries can be directed to the corresponding authors.

## Ethics statement

Written informed consent was obtained from the individual(s) for the publication of any potentially identifiable images or data included in this article.

## Author contributions

YD: Writing – original draft, Writing – review & editing. LS: Writing – review & editing. PL: Writing – review & editing. NL: Writing – review & editing. HZ: Writing – review & editing. BC: Writing – review & editing. HZ: Writing – review & editing. ZT: Writing – review & editing. XZ: Writing – original draft, Writing – review & editing. YZ: Resources, Supervision, Writing – original draft, Writing – review & editing.

## Supplementary material

Supplementary Video 1 Surgical Operation Video during SV-VATS.

Supplementary Video 2 Lung window 3 months after surgery.

Supplementary Video 3 Mediastinal window 3 months after surgery.
